# Supplementary material for: WordCommentsAnalyzer: A windows software tool for qualitative research
Source: F1000Res. 2018 Sep 4;7:536. Originally published 2018 May 3. [Version 2] doi: 10.12688/f1000research.14819.2 (PMC6137411; doi:10.12688/f1000research.14819.2)
Supplement: Supplementary file 3 [file f1000research-7-17614-s0002.tgz › b2a79496-4ada-47c4-9682-a99ce867836d.zip!/Eight Iranian Health Journals/Journal of Knowledge and Health_38.docx]

© Journal of Knowledge and Health. This work is licensed under CC BY 4.0 (https://creativecommons.org/licenses/by/4.0/).

Volume & Issue: دوره 3، شماره 4-3: 1387

Title: طراحی و به¬کارگیری شاخص¬های تخصیص منابع مالی آموزش در دانشگاه¬های علوم پزشکی: کاربرد نظریه «کارفرما – مجری» در مطالعه كيفي

http://knh.shmu.ac.ir/index.php/site/article/view/217

مقدمه: با توجه به اهمیت مديريت اقتصاد در آموزش ضرورت دارد تا به­دنبال راهی برای مدون نمودن علمي چگونگی اختصاص بودجه در دانشکده­های وابسته به دانشگاه­های علوم پزشکی بود. در این پژوهش با استفاده از «نظریه کارفرما – مجری»، پژوهش­گران با هدف تعیین شاخص­هایی برای تخصیص بودجه در دانشکده­های مختلف دانشگاه علوم پزشکی مبتنی به اصول علمی مدیریت و اقتصاد به انجام اين پژوهش اقدام نمودند. مواد و روش­ها: این مطالعه کیفی به­صورت تشکیل جلسات صاحب­نظران و به­روش دلفی انجام گردید. با مطالعه روش­های تخصیص بودجه کشورهای مختلف و با در نظر گرفتن شرایط حاکم بر دانشگاه و دانشکده­های تحت پوشش، شاخص­های تخصیص بودجه تدوین گردیدند. نتایج: تخصیص بودجه آموزش در دانشگاه علوم پزشکی منتخب، متناسب با عملکرد دانشکده­ها، تعداد اعضاء هیأت علمی، تعداد دانشجویان و مقطع دانشجویان و هزینه­بری رشته نبوده است. بررسی­ها نشان داد تخصیص اعتبارات هر دانشکده بیش­تر معطوف به میزان هزینه­های سال قبل و قدرت چانه­زنی رؤسای دانشکده­ها بوده است و برونداد حاصل از عملکرد کم­تر به­عنوان شاخص به­کار برده می­شده است. نتيجه­گيري: بر اساس نتایج به­دست آمده در این پژوهش، اقداماتی نظیر چانه­زنی و توجیهات کلامی برای افزایش سهم دانشکده از بودجه کل دانشگاه در دانشگاه علوم پزشکی منتخب براي انجام این پژوهش جای خود را به ارایه مستندات و عملکرد در جهت توسعه و حتی ارتقاء کیفیت آموزش داد. این مطالعه نشان داد مناسب­ترین شاخص برای تقسیم منابع مالی آموزش بین دانشکده­های مختلف یک دانشگاه، بر اساس تعداد دانشجو و هزینه­بری هر رشته و مقطع می­باشد. در این مطالعه ضرایب هزینه­بری رشته و مقطع تعیین و بومی شد.

Title: طراحي الگوي برنامه¬ريزي عملياتي براي سطح اول شبكه¬هاي بهداشتي درماني ايران

http://knh.shmu.ac.ir/index.php/site/article/view/218

مقدمه: این پژوهش به­منظور طراحي الگوي برنامه­ريزي عملياتي براي سطح اول شبكه­هاي بهداشتي درماني ايران در سال 1387 انجام گرفت. مواد و روش­ها: در این مطالعه کاربردی که به شیوه توصیفی- تطبیقی انجام شده است تعدادی از کشورها و سازمان­های بین المللی دارای نظام فراگیر برنامه­ریزی و تعدادی از سازمان­های منتخب داخلی مورد بررسی قرار گرفتند. داده­های مورد نیاز کشورهای منتخب، از طریق فرم جمع­آوری داده­ها و در سازمان­های داخلی از طریق مصاحبه گردآوری شده و الگوی اولیه بر اساس آن­ها ارایه گردید. الگو از طریق روش دلفی به آزمون گذاشته شده و با نظر خبرگان تغییرات لازم در آن صورت گرفته و الگوی نهایی ارایه گردید. نتایج: کلیه سازمان­های خارجی و منتخب داخلی دارای سیستم برنامه­ریزی بوده ولی هیچ­یک از واحدهای سطح اول مراقبت­های بهداشتی ایران دارای سیستم برنامه­ریزی منسجم نبوده و از الگوی خاصی پیروی نمی­کردند. در وزارت بهداشت درمان و آموزش پزشکی ایران نیز از سال 1387 الگویی نرم­افزاری برای برنامه­ریزی عملیاتی در سطح ستادی مورد استفاده قرار گرفته ولی در سطوح پایین­تر به­خصوص مراکز بهداشتی درمانی شهری و روستایی الگویی وجود نداشت. در کلیه کشورهای منتخب الگوی برنامه­ها شامل عنوان، اهداف یا نتایج مورد انتظار، زمان­بندی، فعالیت­ها و نظام بودجه­بندی و پایش و ارزشیابی بوده ولی در الگوهای رایج کشور ایران گرچه اجزاي يادشده وجود داشت ولی مبناهای علمی در تدوين برنامه­هاي عملياتي به چشم نمي­خورد. مکانیزم طراحی برنامه­ریزی پیشنهادی اولیه دارای 8 مرحله بوده که پس از سه مرحله نظرسنجی خبرگان 7 مرحله مورد تأیید قرار گرفت. نتیجه­گیری: بهره­گیری از الگوی برنامه­ریزی عملیاتی پيشنهادي می­تواند به بهبود شاخص­های عملکردی سطح اول شبکه­های بهداشتی درمانی ایران منجر گردد.

Title: ارزيابی تراکم استخوانی در بيماران دچار سنگ کليه؛ نقش هایپرکلسيوری در از دست رفتن استخوان: آيا محدوديت کلسیم در اين بيماران لازم است ؟

http://knh.shmu.ac.ir/index.php/site/article/view/219

مقدمه: سنگ­های ادراری شکايتی شايع در ارولوژي هستند. در این مطالعه وضعيت تراکم استخوانی در بيماران دچار سنگ­های کلسيمی با گروه شاهد مقایسه شده و هم­چنین تراکم استخوانی مبتلایان به سنگ­های ادراری بر حسب سن، جنس و نوع سنگ بررسی شده است. مواد و روش­ها: در 85 بیمار مبتلا به سنگ ادرای و 85 فرد سالم، متغیرهایی شامل سن، قد، وزن، نمایه توده بدنی، نتایج resco-T وscore -Z حاصل از تراکم سنجی در دو ناحیه­ی کمر و گردن استخوان ران ثبت شد. میزان اوریک اسید، کلسیم، پتاسیم، سدیم، اوره، کراتینین، فسفر، آلکالن فسفاتاز و پاراتیروئید هورمون سرم نيز ارزيابی و ثبت شد. علاوه بر این، مقادير ادرار 24 ساعته هر یک از بيماران مورد مطالعه از جهت کراتینین، اگزالات، سیترات، اسید اوریک، کلسیم، اوره و حجم کلی مورد بررسی قرار گرفت. نتايج: تراکم ماده معدنی استخوان (Bone mineral density) در مهره­های کمری و گردن استخوان ران در بیماران مبتلا به سنگ ادراری به­طور معناداری کم­تر گزارش شده است. این اختلاف پس از طبقه­بندی بیماران و گروه شاهد به دو گروه هايپرکلسيوری و سطح طبیعی کلسيم ادراری نیز معنادار بود. طبق نتایج دانسیتومتری گردن استخوان ران و کمر، کاهش BMD (استئوپنی و استئوپروز) در زنان يائسه به­طور معناداری بیش­تر گزارش شده است. نتیجه­گیری: با توجه به عدم رابطه بین هايپرکلسيوري و از دست دادن استخوان و اين نکته که محدوديت در کلسيم رژيم غذایی نه تنها ايجاد سنگ را کاهش نمي­دهد بلکه باعث بر هم خوردن تعادل کلسيم و از دست رفتن استخوان مي­شود، بنابراین محدوديت کلسيم رژيم غذایی برای بيماران مبتلا به سنگ کليه پيشنهاد نمی­شود.

Title: اندازه¬گيري ميزان بيان ژن MMP-9 برای ارزیابی بهبودی بیماران مبتلا به سرطان پستان

http://knh.shmu.ac.ir/index.php/site/article/view/220

مقدمه: ماتريکس متالو پروتئينازها-9 (MMP-9) آنزيمي با فعاليت پروتئازي است که تاکنون عملکرد آن در ايجاد چندين سرطان گزارش شده است. با توجه به رابطه مقدار فعال اين آنزيم و سرطاني شدن سلول­ها در اين مطالعه ما به بررسي ميزان پلاسمایي اين آنزيم در بهبود يا پيشرفت و متاستاز بيماري سرطان پستان پرداختيم. مواد و روش­ها: در اين مطالعه ميزان بيان MMP-9 در پلاسماي نمونه خون گرفته شده از 98 بيمار داراي سرطان پستان در دو مرحله تشخيص بيماري و بعد از درمان از طريق زيموگرافي کمي ژلاتين (Gelatin quantitative zymography) بررسي شد. نتايج: از کل 80 بيماري که به درمان پاسخ مثبت نشان دادند در 76 نفر غلظت پلاسمایي MMP-9 فعال بعد از درمان کاهش محسوسي نسبت به زمان تشخيص بيماري نشان داد و در 4 نفر از اين بيماران هيچ تغيير در ميزان  MMP-9 پلاسمایي قبل و بعد از درمان مشاهده نشد. هم­چنين در 18 نفر از بيماران که عود مجدد بيماري را نشان دادند (6 نفر سرطان پستان اوليه و 12 نفر متاستاز به ديگر نواحي) اين ميزان نسبت به قبل از درمان افزايش يافته بود. نتيجه­گيري: يافته­هاي ما نشان­دهنده وجود ارتباط بسيار مثبت بين سطح پلاسمایي اين آنزيم و بهبود يا بدخيمي سرطان پستان در گروه مورد مطالعه است و طبق يافته­هاي ما ميزان MMP-9 فعال پلاسمایي مي­تواند به­عنوان عاملی سودمند براي بررسي وضعيت پاسخ بيماران سرطان پستان به درمان، مورد استفاده قرار گيرد.

Title: بررسی اضطراب امتحان و ارتباط آن با عملکرد تحصیلی در دانشجويان پرستاري

http://knh.shmu.ac.ir/index.php/site/article/view/221

مقدمه: اضطراب امتحان یک پدیده شایع در میان دانشجویان و از جمله مشکلات نظام آموزشی محسوب می­شود. با توجه به تأثیر اضطراب امتحان بر عملکرد تحصیلی افراد این مطالعه با هدف تعیین اضطراب امتحان و ارتباط آن با عملکرد تحصیلی در دانشجويان پرستاري انجام شده است. مواد و روش­ها: مطالعه حاضر یک مطالعه توصیفی- تحلیلی می­باشد که بر روی 150 نفر از دانشجویان پرستاری مشغول به تحصیل در دانشکده پرستاری آبادان در سال 1386 انجام شده است. ابزار گردآوری داده­ها شامل پرسش­نامه­ای مشتمل بر دو قسمت: ویژگی­های دموگرافیک و پرسش­نامه اضطراب امتحان (Test Anxiety Inventory) بوده که به­روش خود ایفا در زمان برگزاری امتحانات پایان ترم تکمیل گردیدند. تجزیه و تحليل داده­های پژوهش با استفاده از شاخص­های مرکزی و پراکندگی و آزمون­ من ویتنی و نیز ضریب هم­بستگی اسپیرمن توسط نرم­افزار 13.0 SPSS انجام شد. نتایج: ميانگين نمره اضطراب امتحان دانشجويان در اين پژوهش 2/23± 9/30 بود. 14% دانشجویان اضطراب امتحان نداشتند، 7/48% اضطراب كم، 34% اضطراب متوسط و 3/3% اضطراب امتحان زياد داشتندکه در مجموع 86% از دانشجویان دارای درجاتی از اضطراب امتحان بودند. در این مطالعه ارتباط معنادار آماری بین اضطراب امتحان با عملکرد تحصیلی (معدل كل) (13/0=P)، سن (84/0=P)، وضعیت تأهل (053/0=P) و مقطع تحصیلی دانشجویان(057/0=P) به­دست نیامد. نتیجه­گیری: نظر به شیوعٌ بالای اضطراب امتحان در دانشجویان، توصیه می شود ضمن توجه ویژه به این پدیده، پژوهش­های بیش­تری جهت شناسایی عوامل مرتبط با آن و ارایه­ی راهکارهایی جهت کاهش آن در دانشجویان انجام گیرد.

Title: بررسی تأثیر رنگ در فیلم¬های آموزشی بر میزان یادگیری

http://knh.shmu.ac.ir/index.php/site/article/view/222

مقدمه: مواد آموزشی مبتنی بر تأثیرات روانشناختی باید هم از نظر عناصر مجزا و هم ترکیب قدرت تخیل را بپروراند، حواس را تقویت کند و برانگیزانند، باشد. هدف این پژوهش بررسی تأثیر رنگ بر میزان یادگیری در فیلم­های آموزشی می­باشد. مواد و روش­ها: در این پژوهش از میان 1777 دانش­آموز پایه چهارم مدارس دولتی شاهرود به­روش نمونه­گیری خوشه­ای تصادفی 8 کلاس (241 دانش­آموز) و از فصول کتاب علوم 4 فصل انتخاب شده و 8 فیلم در رابطه با محتوا به­صورت سیاه و سفید و رنگی تولید و پس از نمایش فیلم­ها، آزمونی حاوی 10 سؤال بر مبنای محتوای فیلم­های آموزشی گرفته شد. پس از آزمون، میانگین نمرات با استفاده از آزمون t مستقل تجزیه و تحلیل شد. هم­چنین 30 نفر از دانش­آموزان یک ماه بعد از آزمون اولیه، مجدد به­منظور بررسی پایداری یادگیری به وسیله فیلم­های رنگی مورد آزمون قرار گرفتند. نتایج: نتایج نشان داد که میانگین نمرات دانش­آموزانی که فیلم رنگی دیده­اند برابر با 68/3 و در دانش­آموزانی که فیلم سیاه و سفید دیده­اند برابر با 39/3 می­باشد که اختلاف آن­ها معنادار می­باشد. در بررسی یک ماه بعد از نمایش فیلم­های رنگی، اختلاف معناداری بین میانگین نمرات آزمون در دو مقطع زمانی مشاهده نشد. نتیجه­گیری: امروزه با توجه به نیاز روزافزون مراکز آموزشی فنی، علمی و دانشگاهی به رسانه­های آموزشی و از طرفی دسترسی آسان و ارزان به انواع ابزارهای تولید فیلم که حتی قابل نصب در کامپیوترهای شخصی می­باشد، پرداختن به اختصاصات فیلم­های آموزشی ضروری به نظر می­رسد. لذا پیشنهاد می­شود در تولید رسانه­های آموزشی استفاده از انواع رنگ­ها به­طور علمی، مورد توجه کارشناسان قرار گیرد.

Title: بررسي تأثیر سروصداي مداوم و متناوب بر عملكرد حل مسأله دانشجويان

http://knh.shmu.ac.ir/index.php/site/article/view/223

مقدمه: سروصدا يکي از مهم­ترين منابع فشار‌هاي زندگي محسوب مي‌شود به­طوري­كه نتايج حاصل از پژوهش­هاي محققين نشان داده است که صداي مزاحم علاوه بر اينكه از نظر جسماني مي­تواند به کاهش شنوايي، افزايش فشارخون و افزايش آسيب­پذيري منجر گردد، يک عامل استرس­زاي محيطي بوده و حوزه‌ي شناخت و عملکرد شناختي را نيز متأثر مي­نمايد به­نحوي كه سبب کم شدن دقت در فعاليت­هاي مغزي و ناهماهنگي درکارهاي فکري و فهم مطالب مي‌گردد، اين ناهماهنگي و بي­دقتي به­خصوص در زمينه‌ي راهبردهاي حل تکليف و منحرف کردن توجه از جنبه‌هاي متفاوت آن بيش­تر مشهود است. مواد و روش­ها: اين مطالعه تجربي بر روي 36 نفر از دانشجويان دوره­ي کارشناسي دانشگاه الزهراء (س) که دامنه‌ي سني آن­ها بين 19تا 24 سال است، انجام گرفت و نمرات دانشجويان در آزمون استدلال منطقي آيزنك، هنگامي كه در معرض سروصداي ترافيك تهران قرار گرفته بودند، مبناي محاسبه قرار گرفت. میانگین نمرات آزمون استدلال آیزنک با استفاده از آزمون آنالیز واریانس یک­طرفه با هم مقایسه شده است. نتايج: نتايج نشان داد بين ميانگين­هاي عامل تکليف پيچيده­ي گروه­هاي پژوهش تفاوت معناداري وجود دارد. بدين معنا كه گروهي از دانشجويان كه در معرض سروصداي متناوب قرار گرفته بودند در حل مسايل پيچيده، عملكرد ضعيف­تري نسبت به سايرين داشتند. نتيجه­گيري: نتايج مطالعه نشان مي­دهد تكاليف شناختي پيچيده از سروصداي غير قابل پيش­بيني اثر مي­پذيرد لذا بايد براي كنترل سروصداي محيط­هاي كاري، فكري و آموزشي كه در آن افراد در معرض اين نوع سروصدا قرار دارند و هم­زمان به فعاليت­هاي شناختي مشغولند، تدابيري انديشيده شود.

Title: بررسي شيوع فشارخون بالا و برخي عوامل مؤثر بر آن در يک نمونه از جمعيت روستايي با تنوع قومي

http://knh.shmu.ac.ir/index.php/site/article/view/224

مقدمه: اين مطالعه به­منظور تعيين شيوع فشارخون بالا و عوامل مؤثر بر آن در يک نمونه از جمعيت روستایي بالاي 30 سال ايران که علاوه بر ساختار روستايي از تنوع قومي نيز برخوردار است، انجام شده است. مواد و روش­ها: مطالعه به­روش مقطعي و با حجم نمونه 1500 نفر و با روش نمونه­گيري خوشه­اي در جمعیت روستایی شهرستان آق­قلا  انجام شد. داده­ها با توجه به ماهيت متغيرها از طريق مصاحبه، اندازه­گيري و مشاهده گردآوري گردید. از روش رگرسيون لجستيک براي آناليز داده­ها استفاده شد. نتایج: شيوع فشارخون بالا به­طورکلي 4/18 درصد برآورد گرديد و زنان بيش­تر از مردان مبتلا به اين بيماري بودند و شيوع آن در قوميت ترکمن بيش­تر از اقوام سيستاني و بلوچ بود. فشارخون بالا با چاقي و کم­تحرکي رابطه معنادار داشت و با افزايش سن، شيوع فشارخون روند افزايشي نشان داد. عوامل خطر عمده براي فشارخون بالا عبارت بودند از: سن بالاي 60 سال، BMI بالا، عدم تحرك فيزيكي و نسبت بالاي دور شكم به دور باسن. نتيجه­گيري: شيوع بيش­تر پرفشاري خون در قوميت تركمن در درجه اول مربوط به افزايش وزن و BMI بالا و در درجه دوم ناشي از عدم تحرك فيزيكي كافي بوده است. فراواني بيش­تر عوامل خطر در زنان نسبت به مردان شانس ابتلا به فشارخون را افزايش داده بود نه جنسيت آن­ها.

Title: ارتباط بین شاخص¬هاي تن¬سنجي با سن بلوغ دانش¬آموزان دختر 14-11 ساله شاهرود

http://knh.shmu.ac.ir/index.php/site/article/view/226

مقدمه: سوء تغذیه دوران کودکی آغاز بلوغ را به تأخیر می­اندازد ولی از وقوع آن پیش­گیری نمی­کند. هدف این مطالعه بررسی ارتباط بین شروع قاعدگی با شاخص­های تن­سنجی نوجوانان 11 تا 14 ساله می­باشد. مواد و روش­ها: در این مطالعه توصيفي- تحليلي، 418 دانش­آموز دختر مقطع راهنمائی (11 تا 14 ساله) در شهر شاهرود مورد برسی قرار گرفتند. نمونه­ها به­طور تصادفی ساده انتخاب و برای نشان دادن وضعیت تغذیه دانش­آموزان از شاخص­هاي تن­سنجی قد و وزن برای سن، وزن، قد و شاخص نمايه توده بدن استفاده و سپس با استاندارد NCHS مقایسه شده است. نتایج: در این مطالعه ميانگين BMI در دختران قاعده شده 1/3±8/19 و در دختران قاعده نشده 0/3±6/17 بوده است. میانگین سن دختران قاعده شده 8/0±9/12 سال بوده و بین سن و شروع قاعدگی ارتباط معناداری مشاهده گردید (0001/0>P). 47% دختران در تابستان به بلوغ رسیده­اند. میانگین وزن و قد دانش­آموزان قاعده شده به­ترتیب 5/8±7/48 کیلوگرم و 7/6±9/156 سانتی­متر بوده است. بین وزن، قد، شاخص توده بدن و تن­سنجي دانش­آموزان و وضعیت قاعدگی ارتباط معناداری دیده شد (0001/0=P). با استفاده از آنالیز آماري تفاوت معناداری بین وضعيت قاعدگي با وضعيت اجتماعي و اقتصادي ملاحظه گردید. نتيجه­گيري: نتايج مطالعه نشانگر آن است كه بین وضعیت تغذیه و سن بلوغ ارتباط معناداری وجود دارد. با توجه به اهمیت سن بلوغ پایین آمدن سن منارک و ایجاد میل جنسی و تأثیر شاخص­هاي نمايه توده بدن و تن­سنجي با شروع قاعدگی توجه به وزن ایده­آل و تغذیه مناسب، تحرك كافي جهت اين گروه آسیب­پذیر توصیه می­گردد.
